# Phase II study of docetaxel and irinotecan combination chemotherapy in metastatic gastric carcinoma

**DOI:** 10.1038/sj.bjc.6603133

**Published:** 2006-04-25

**Authors:** S R Park, J H Chun, M S Yu, J H Lee, K W Ryu, I J Choi, C G Kim, J S Lee, Y W Kim, J-M Bae, H K Kim

**Affiliations:** 1 Research Institute & Hospital, National Cancer Center, 809 Madu1, Ilsan, Goyang, Gyeonggi 410-769, Republic of Korea

**Keywords:** docetaxel, gastric cancer, irinotecan

## Abstract

The current treatment for metastatic gastric cancer (MGC) consists of cisplatin and/or fluorouracil (5-FU) based combination chemotherapy, but cisplatin-based regimens are associated with considerable toxicity. We evaluated the efficacy and safety of a noncisplatin-, non-5-FU-containing regimen, docetaxel/irinotecan in MGC. Chemo-naive patients with MGC received docetaxel (30 mg m^−2^) and irinotecan (70 mg m^−2^) on days 1 and 8 every 3 weeks. The 48 eligible patients (median age 56 years) received a median of four cycles of docetaxel/irinotecan (range 1–18). Of the 46 patients in whom efficacy could be evaluated, 21 showed a partial response (response rate=45.7%; 95% confidence interval (CI) 31.3–60.1%). At a median follow-up of 15.0 months, the median time to progression was 4.5 months (95% CI 3.8–5.2 months) and overall survival was 8.2 months (95% CI, 5.8–10.6 months). Grade 3/4 neutropenia developed in 57.4% of patients, and febrile neutropenia/neutropenic infection in 19.1%. Nonhaematological toxicities were moderate; grade 3/4 diarrhoea occurred in 19.1% of patients, however, was manageable by a dose reduction. There was one possible treatment-related death. In conclusion, weekly docetaxel/irinotecan is a promising outpatient regimen in MGC, with appropriate dose modification.

Despite its decreasing incidence over the past few decades, gastric cancer remains one of the major causes of cancer deaths worldwide (Jemal *et al*, 2004) and is the most prevalent malignancy in Korea, constituting 24% of all solid tumours in males and 15% in females (Shin *et al*, 2004). Systemic chemotherapy is often used in patients with locally advanced or metastatic gastric cancer (MGC), but results of most combination regimens have been unsatisfactory, with median survival times of 6–9 months (Webb *et al*, 1997; Vanhoefer *et al*, 2000; Ohtsu *et al*, 2003). To data, the combination chemotherapies most commonly used have been based on fluorouracil (5-FU) and/or cisplatin, with 5-FU/cisplatin (FP) and epirubicin/cisplatin/5-FU currently regarded as reference treatment. However, cisplatin-based chemotherapy is associated with an unfavuorable toxicity profile, including severe emesis, neurotoxicity and nephrotoxicity. In addition, the need for intense intravenous (i.v.) hydration complicates the administration of this drug. Therefore, there is a need to develop active but less toxic chemotherapy regimens, which include new active compounds.

Docetaxel, which inhibits microtubule depolymerization, has been widely used in the treatment of MGC, with several phase II trials showing that this drug, as a single agent, induces response rates of 16–24% (Sulkes *et al*, 1994; Bang *et al*, 2002). Moreover, when combined with cisplatin, docetaxel has produced response rates of 33–56% (Roth *et al*, 2000; Kettner *et al*, 2001; Ridwelski *et al*, 2001; Ajani *et al*, 2005).

Irinotecan, which inhibits topoisomerase I, thereby disrupting DNA replication, is a cytotoxic agent with promising activity. In MGC, irinotecan monotherapy has shown response rates of 20–23% (Futatsuki *et al*, 1994; Kohne *et al*, 2003) and irinotecan plus cisplatin has resulted in response rates of 32–58% (Ajani *et al*, 2002; Lim *et al*, 2003; Pozzo *et al*, 2004).

Given the activity of docetaxel and irinotecan in MGC and the different mechanisms of the two drugs, we designed a phase II trial to examine the efficacy and toxicity of this new combination in patients with MGC. As previous study of every-3-weeks docetaxel/irinotecan in the chemo-naïve patients with advanced gastric cancer showed considerable toxicities, especially grade 3/4 neutrpenia (85.4%) and febrile neutropenia/neutropenic infection (41.4%) (Hawkins *et al*, 2003), and both drugs are myelosuppressive, we administered each of these drugs once per week.

## PATIENTS AND METHODS

### Patients

Patients eligible for this trial were those aged over 18 years old with histologically proven gastric adenocarcinoma, metastatic disease, unidimensionally measurable disease, no prior chemotherapy including adjuvant chemotherapy, Eastern Cooperative Oncology Group (ECOG) performance status 0–2, and adequate baseline hematological function (absolute neutrophil count (ANC)⩾1. 5 × 10^9^ l^−1^, platelet count ⩾100 × 10^9^ l^−1^), hepatic function (serum aspartate aminotransferase and alanine aminotransferase ⩽2.5 × the upper normal limit (UNL), serum bilirubin ⩽UNL) and renal function (serum creatinine ⩽UNL).

Patients were excluded if they had a history of other malignancies within the previous 3 years or pre-existing peripheral neuropathy of grade ⩾2 on the basis of the National Cancer Institute Common Toxicity Criteria (NCI CTC) (version 2.0). Written informed consent was obtained for all patients, and the institutional review board approved the protocol.

### Treatment

Docetaxel (30 mg m^−2^) was administered as a 1-h i.v. infusion and irinotecan (70 mg m^−2^) as a 90-min i.v. infusion on days 1 and 8 of each 3-week cycle. Oral dexamethasone (8 mg twice daily for six doses, starting 24 h before docetaxel) and parenteral pheniramine maleate (45.5 mg) were administered prophylactically. Prophylactic administration of granulocyte-colony stimulating factor (G-CSF) was not allowed. The next chemotherapy cycle was delayed if ANC fell below 1.5 × 10^9^ l^−1^, the platelet count below 100 × 10^9^ l^−1^ or if patient experienced any other nonhaematological toxicity excluding alopecia greater than grade 1. Doses of docetaxel and irinotecan were reduced by 20% in subsequent cycles if patients experienced grade 3 or 4 neutropenia with fever, grade 4 thrombocytopenia, or any grade ⩾3 nonhaematological toxicity. The dose of docetaxel was reduced by 20% in subsequent cycles if patients experienced grade 2 or 3 neurologic toxicity or recurrent fluid retention. The dose of irinotecan was reduced by 20 and 40% in patients experiencing grade 2 diarrhoea and 3 or 4 diarrhoea, respectively. Chemotherapy was administered on an outpatient basis and continued until disease progression or unacceptable toxicity developed.

### Assessment of efficacy and toxicity

During the first two cycles, complete blood cell count and blood chemistry was performed weekly. In subsequent chemotherapy cycles, these tests were performed on days 1 and 8. A computed tomography (CT) scan was performed every three cycles to evaluate response to treatment. Complete response (CR), partial response (PR), stable disease (SD) and progressive disease (PD) were defined according to the response evaluation criteria in solid tumour (RECIST) (Therasse *et al*, 2000). Responses were confirmed by an expert independent radiologist. Time to progression (TTP) was calculated from the date of first chemotherapy cycle to the date of disease progression, death from any cause, or last follow-up. Overall survival (OS) was calculated from the date of first chemotherapy cycle to the date of death from any cause or last follow-up. Toxicity was assessed before drug dosing using the NCI CTC (version 2.0).

### Statistical analysis

The primary end point of this study was response rate. Secondary end points were TTP, OS and safety. The optimal Simon two-stage design was used to determine the sample size (Simon, 1989). An interim analysis was carried out when the first 15 assessable patients had been recruited. If there were four or fewer responses, the study was to be terminated. Otherwise, accrual was to be continued to a total of 46 patients. If there were more than 18 responses among 46 patients, the treatment was considered sufficiently active with a significance level of 5% and power of 80%. TTP and OS were analysed according to the Kaplan–Meier method and updated to 30 August 2005.

## RESULTS

### Patient characteristics

From December 2003 to September 2004, 49 patients were enrolled in this study, 29 (59.2%) males and 20 (40.8%) females, of median age 56 years (range 29–72 years). Most of the patients (98.0%) had ECOG PS 0-1, and 26 (53.1%) had multiple metastases involving two or more organ systems. Metastatic sites were in the abdominal lymph nodes (51.0%), peritoneum (49.0%), liver (36.7%), ovary (12.2%) and others (34.7%) (Table 1). All patients had metastatic disease at diagnosis and two of them received palliative gastrectomy before the chemotherapy.

### Efficacy

**Figure 1:**
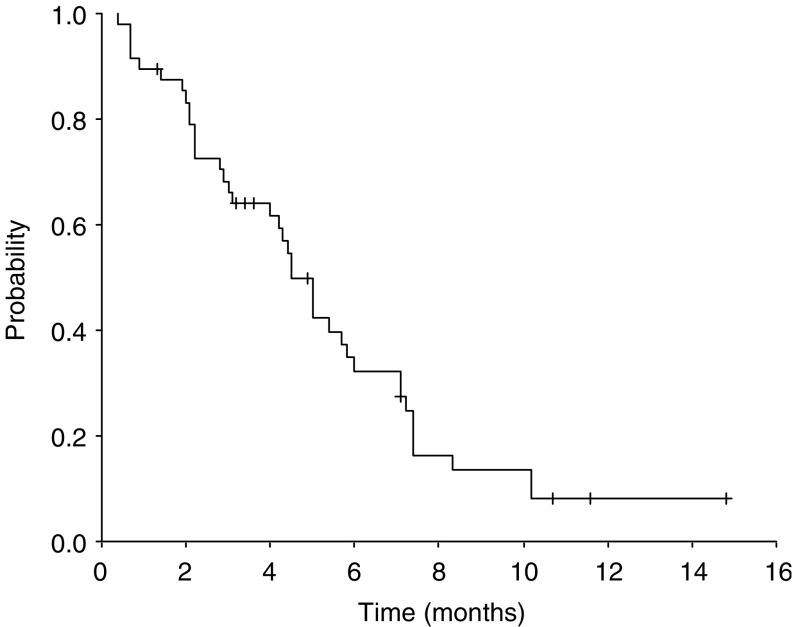
Time to progression for all eligible patients.

**Figure 2:**
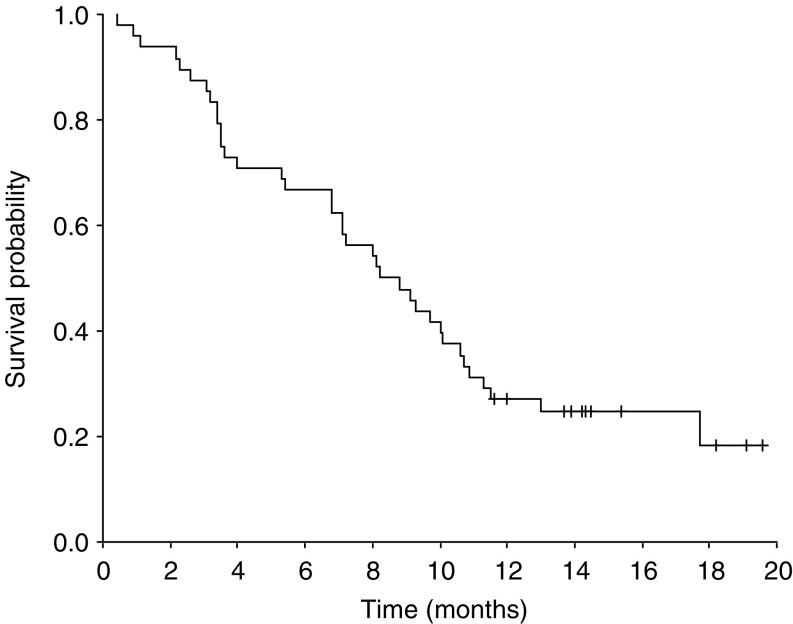
Overall survival for all eligible patients.

Of the 49 patients, 46 could be evaluated for response, whereas one was ineligible because of a previous history of cervical cancer and two were lost to follow-up prior to receiving three cycles of chemotherapy. According to the independent review panel, 21 patients (45.7%) achieved confirmed PR, 15 (32.6%) had SD and 10 (21.7%) had PD. Thus, the overall response rate was 45.7% (95% confidence interval (CI) 31.3–60.1%) (Table 2). All objective responses were confirmed by follow-up CT at least 4 weeks after the initial documentation of PR. The median duration of response was 4.8 months (range 1.8–11.5+ months). The median follow-up time was 15.0 months (range 11.6–21.0 months), during which the median TTP was 4.5 months (95% CI 3.8-5.2 months) (Figure 1) and the median OS was 8.2 months (95% CI 5.8–10.6 months) (Figure 2). The 1-year survival rate was 27.1% (95% CI 14.6–39.6%).

### Treatment delivered

A total of 250 cycles were administered, with a median of 4 per patient (range 1–18). Treatment was delayed for a median of 6 days (range 4–28 days) in 31 cycles (12.4%) in 21 patients (43.8%), mainly because of febrile neutropenia/infection with neutropenia (seven cycles). Fourteen cycles were delayed due to reasons unrelated to disease or treatment, including pending imaging studies to evaluate response or the patient's request. Dose reduction was required in 126 (52.4%) cycles in 26 patients (54.2%), primarily due to diarrhoea (17 patients (35.4%)) and febrile neutropenia/infection with neutropenia (11 (22.9%)). The relative dose intensities of docetaxel and irinotecan were 84.0% (16.7 mg m^−2^ per week) and 79.3% (37.0 mg m^−2^ per week), respectively.

Of the 48 eligible patients, 35 (72.9%) were discontinued due to disease progression, 2 (4.2%) due to cerebral infarction, 1 (2.1%) each for recurrent grade 3 diarrhoea, pulmonary embolism, gastric tumour bleeding and death. During the study, seven patients (14.6%) withdrew their consent because of grade 1 or 2 fatigue (*n*=4) and other reasons, including referral to other hospital (*n*=1) and economic problem (*n*=2).

### Toxicity

Forty-seven patients were assessable for toxicity. Table 3 summarizes chemotherapy toxicities per patient. The most common haematological toxicity was neutropenia, which occurred at grade 3/4 intensity in 27 patients (57.4%). Febrile neutropenia occurred in seven patients (14.9%) and infection with grade 3/4 neutropenia in four patients (8.5%). All of these patients were successfully treated with antibiotics and G-CSF. Grade 3/4 thrombocytopenia and anaemia occurred in one (2.1%) and seven (14.9%) patients, respectively.

Nonhaematological toxicities were mild to moderate and manageable. The most common grade 3/4 nonhaematological toxicity was diarrhoea (19.1%), followed by infection without neutropenia (10.6%), nausea (8.5%), stomatitis (6.4%), anorexia (4.3%), fatigue (2.1%), vomiting (2.1%) and docetaxel-induced pneumonitis (2.1%). There was one (2.1%) possible treatment-related death; this patient died at home of unknown causes 5 days after the last dose of chemotherapy in a cycle.

### Second-line chemotherapy

During the follow-up period, 27 (56.3%). of the 48 eligible patients received second-line chemotherapy, mostly containing cisplatin. Of the 27 patients, 15 received FP, six received capecitabine/cisplatin, three received oxaliplatin/5-FU/leucovorin and three received other regimens. Of the efficacy-evaluable 23 patients, three (13.0%) achieved PR, six (26.1%) had SD and 14 (60.9%) showed progression. The median TTP of second-line chemotherapy was 2.4 months (95% CI 1.7–3.1 months).

## DISCUSSION

We have shown here that weekly docetaxel in combination with weekly irinotecan is an active first-line chemotherapy regimen for MGC. The overall response rate of 45.7%, median TTP of 4.5 months and OS of 8.2 months were comparable with previously published regimens, most of which are cisplatin-based. (Roth *et al*, 2000; Kettner *et al*, 2001; Ridwelski *et al*, 2001; Ajani *et al*, 2002; Lim *et al*, 2003; Pozzo *et al*, 2004; Ajani *et al*, 2005; Dank *et al*, 2005; Moiseyenko *et al*, 2005).

The administration of docetaxel-irinotecan every 3 weeks was performed as first-line chemotherapy for this disease with response rate of 37.5% (Hawkins *et al*, 2003). Although our weekly regimen showed lower incidence of grade 3/4 toxicities than tri-weekly regimen (neutropenia 57.4 *vs* 85.4%, febrile neutropenia/infection with neutropenia 23.4 *vs* 41.4%, and diarrhoea 19.1 *vs* 42.9%), its toxicity should be generally considered significant. Cisplatin-containing regimens have shown grade 3/4 neutropenia, febrile neutropenia/infection with neutropenia and diarrhoea in 27–87%, 2–30% and 5–22% of patients, according to the published literature. On the other hand, compared with cisplatin-containing regimens, docetaxel/irinotecan combination was associated with a lower incidence of grade 2 or higher neurotoxicity (2.1 *vs* 2.0-30.8%) and all grade ototoxicity (0 *vs* 52.7%) (Roth *et al*, 2000; Kettner *et al*, 2001; Ridwelski *et al*, 2001; Ajani *et al*, 2002; Lim *et al*, 2003; Pozzo *et al*, 2004; Ajani *et al*, 2005; Dank *et al*, 2005; Moiseyenko *et al*, 2005). In addition, this regimen did not resulted in any grade of nephrotoxicity.

Notably, this weekly regimen involved frequent dose reduction, especially of irinotecan, primarily due to diarrhoea, which occurred mainly in the first 1–3 cycles of therapy and was hardly repeated subsequently after a dose reduction. So, we would propose that the recommended irinotecan dose of this weekly regimen be lowered from 70 to 60 mg m^−2^, and that this regimen should be given only to patients with good performance status without any comorbidities. Yet, this weekly regimen allowed us to better modify subsequent drug dose and better manage drug toxicity with at least comparable therapeutic efficacy, compared with tri-weekly regimen. Taken together, we conclude that weekly docetaxel and irinotecan combination chemotherapy is a promising outpatient regimen in MGC, with appropriate dose modifications.

## Figures and Tables

**Table 1:** Patient characteristics

**Characteristics**	**Number of patients (%)**
Number of entered patients	49
Number of eligible patients	48
	
*Age (years)*	
Median	56
Range	29–72
	
*Gender*	
Male	29 (59.2)
Female	20 (40.8)
	
*ECOG performance status*	
0	3 (6.1)
1	45 (91.9)
2	1 (2.0)
	
*Hiostology*	
Adenocarcinoma, well differentiated	2 (4.1)
Adenocarcinoma, moderately differentiated	14 (28.6)
Adenocarcinoma, poorly differentiated	17 (34.7)
Signet ring cell carcinoma	16 (32.6)
	
*Metastatic organ sites*	
Abdominal lymph node	25 (51.0)
Peritoneum	24 (49.0)
Liver	18 (36.7)
Ovary	6 (12.2)
Others^a^	17 (34.7)
	
*Number of metastatic organ sites*	
1	23 (46.9)
2	14 (28.6)
⩾3	12 (24.5)

ECOG=Eastern Cooperative Oncology Group.

a Cervical lymph node, lung, bone, adrenal gland, uterus, gall bladder, pancreas.

**Table 2:** Response to chemotherapy of 46 evaluable patients

**Response**	**Number of patients (%)**
Complete response	0 (0)
Partial response	21 (45.7)
Stable disease	15 (32.6)
Progressive disease	10 (21.7)
Overall response rate (%)^a^	45.7
95% CI (%)	31.3–60.1

a Overall response=complete response+partial response. CI=confidence interval.

**Table 3:** Toxicity of chemotherapy per patient (*n*=47)

	**NCI-CTC Grade (%)**			
**Toxicity**	**1**	**2**	**3**	**4**
*Haematological*				
Leukopenia	5 (10.6)	11 (23.4)	16 (34.0)	3 (6.4)
Neutropenia	2 (4.3)	5 (10.6)	16 (34.0)	11 (23.4)
Febrile neutropenia	—	—	7 (14.9)	0 (0)
Thombocytopenia	0 (0)	0 (0)	1 (2.1)	0 (0)
Anaemia	11 (23.4)	27 (57.4)	7 (14.9)	0 (0)
				
*Nonhaematological*				
Stomatitis	14 (29.8)	8 (17.0)	3 (6.4)	0 (0)
Anorexia	21 (44.7)	25 (53.2)	2 (4.3)	0 (0)
Nausea	28 (59.6)	14 (29.8)	4 (8.5)	—
Vomiting	12 (25.5)	21 (44.7)	1 (2.1)	0 (0)
Diarrhea	17 (36.2)	11 (23.4)	7 (14.9)	2 (4.3)
Constipation	17 (36.2)	3 (6.4)	0 (0)	0 (0)
Fatigue	26 (55.3)	14 (29.8)	1 (2.1)	0 (0)
Tearing	4 (8.5)	0 (0)	0 (0)	—
Myalgia	1 (2.1)	1 (2.1)	0 (0)	0 (0)
Alopecia	21 (44.7)	23 (48.9)	—	—
Fluid retention	8 (17.0)	6 (12.8)	0 (0)	
Peripheral neuropathy	20 (42.6)	1 (2.1)	0 (0)	0 (0)
Abnormal liver function	12 (25.5)	4 (8.5)	0 (0)	0 (0)
Pneumonitis	0 (0)	1 (2.1)	0 (0)	1 (2.1)
				
*Infection*				
With neutropenia	—	—	3 (6.4)	1 (2.1)
Without neutropenia	0 (0)	0 (0)	5 (10.6)	0 (0)

NCI-CTC=National Cancer Institute Common Toxicity Criteria.
